# Supplementary material for: Patterns of Intron Gain and Loss in Fungi
Source: PLoS Biol. 2004 Nov 30;2(12):e422. doi: 10.1371/journal.pbio.0020422 (PMC532390; doi:10.1371/journal.pbio.0020422)
Supplement: Table S1 — Also available at http://genes.mit.edu/NielsenEtAl/. (4.3 MB ZIP). [file pbio.0020422.st001.zip › NielsenEtAl/html/80.html]

AN5886.1.NCU04385.1.MG01553.1.FG09589.1


```
 CLUSTAL W (1.82) Multiple Sequence Alignments - Introns Inserted


Sequence 1: NCU04385.1	779 aa
Sequence 2: MG01553.1	778 aa
Sequence 3: FG09589.1	777 aa
Sequence 4: AN5886.1	772 aa
Alignment Length: 782 aa
Number Identitical Residues: 511 aa
Alignment Score (without introns) 23059


MG01553.1 	MPGAESTPQTLYDKVLQAHVVDEKLDGTVLLYID1RHLVHEVTSP0QAFEGLRNAGRKVR
NCU04385.1	MPSAESTPRTLYDKVFQAHIVDEKLDGTILLYID~RHLVHEVTSP0QAFEGLENAGRQVR
FG09589.1 	MPAPVGTPQTLYDKVFSAHIVDEKLDGTILLYID1RHLVHEVTSP0QAFEGLENAGRKVR
AN5886.1  	MPGADRKPKTLYDKVFDHHIVNEQEDGTVLIYID1RHLVHEVTSP0QAFEGLKNANRKVR
          	**..  .*:******:. *:*:*: ***:*:*** ********** ******.**.*:**

MG01553.1 	RPDCTLATTDH0NVPTTSRKALKDIASFIKEDDSRTQCVTLEENVKEFGVTYFGLSDKRQ
NCU04385.1	RPDCTLATTDH0NVPTTSRKALKDIASFIKEDDSRTQCVTLEENVKKFGITYFGLSDKRQ
FG09589.1 	RPDCTLATTDH0NVPTSSRKALKDIASFIEEDDSRTQCVTLEENVKKFGITYFGLGDKRQ
AN5886.1  	RPDCTLVTVDH0NIPTSSRKNFKNVEQFIEENDSRLQCSTLEENVKDFGLTYFGMDDKRQ
          	******.*.** *:**:*** :*:: .**:*:*** ** *******.**:****:.****

MG01553.1 	GIVHVIGPEQGFTLPGTTVVCGDSHTSTHGAFGALAFGIGTSEVEHVLATQCLITKRSKN
NCU04385.1	GIVHVIGPEQGFTLPGTTVVCGDSHTSTHGAFGALAFGIGTSEVEHVLATQCLITKRSKN
FG09589.1 	GIVHIIGPEQGFTLPGTTVVCGDSHTSTHGAFGALAFGIGTSEVEHVLATQCLITKRSKN
AN5886.1  	GIVHVIGPEQGFTLPGTTVVCGDSHTSTHGAFGALAFGIGTSEVEHVLATQTLITRRSKN
          	****:********************************************** ***:****

MG01553.1 	MRIQVDGELAPGVSSKDVVLHAIGIIGTAGGTGAVIEFCGSVIRSLSMEARMSICNMSIE
NCU04385.1	MRVQVDGELAPGVSSKDVILHVIGQIGTAGGTGAVIEFCGSVIRSLSMEARMSICNMSIE
FG09589.1 	MRIQVDGELAPGVSSKDIVLHAIGQIGTAGGTGAVIEFCGSVIRSLSMEARMSICNMSIE
AN5886.1  	MRVQVDGELPAGVTSKDVVLHIIGLIGTAGGTGCVIEFCGSVIRGLSMEARMSMCNMSIE
          	**:******..**:***::** ** ********.**********.********:******

MG01553.1 	GGARAGMVAPDEITFEYLKGRPLAPKYDSPEWHKATQYWKNLQSDPGAKYDIDVFIDAKD
NCU04385.1	AGARAGMVAPDEITFEYLKGRPLAPRYGSEEWNKAVTYWKSLASDPDAKYDIDVFIDGKD
FG09589.1 	GGARAGMVAPDEITFEYLKGRPLAPKYGSETWNNAVAYWKSLQSDPGAKYDIDVFIDAKD
AN5886.1  	GGARAGMVAPDETTFEYLKGRPLAPKYDSAEWKKAVSYWSSLASDEDAVYDKTILIDAKD
          	.*********** ************:*.*  *::*. **..* ** .* **  ::**.**

MG01553.1 	IVPTLTWGTSPEDVVPITGVVPDPETFATEAKKADGRRMLQYMGLKAGTPMEDIPVDKVF
NCU04385.1	IIPTVTWGTSPEDVVPITGVVPDPETFATEGKKAAGRRMIEYMGLVPGTPMEDIEVDKVF
FG09589.1 	IVPTLTWGTSPEDVVPITGCVPDPETFSSESKKAAGRRMLEYMGLTAGTPMEDIPVDKVF
AN5886.1  	IVPTISWGTSPQDVVPITGVVPGPDDFEDEARKAACKRALEYMGLTAGTPMKDVTVDKVF
          	*:**::*****:******* **.*: *  *.:**  :* ::**** .****:*: *****

MG01553.1 	IGSCTNSRIEDLRAAAAVVKGRKKAPNVKSAMVVPGSGLVKTQAEEEGLDKIFEEAGFEW
NCU04385.1	IGSCTNSRIEDLRAAAQVVKGKKIAANIKRALIVPGSGLVKDQAEAEGLDKIFQEAGFEW
FG09589.1 	IGSCTNARIEDLRAAANVVKGRKVADNIKRAMVVPGSGLVKAQAEEEGLDQIFVDAGFEW
AN5886.1  	IGSCTNSRIEDLRAAANVVRGKKVASNIKRAMVVPGSGLVKQQAEAEGLDKIFIDAGFEW
          	******:********* **:*:* * *:* *::******** *** ****:** :*****

MG01553.1 	REAGCSMCLGMNPDILAPQERCASTSNRNFEGRQGAGGRTHLMSPVMAAAAGIVGKLADV
NCU04385.1	REAGCSMCLGMNPDILSPKERCASTSNRNFEGRQGAQGRTHLMSPVMAAAAAIVGKLADV
FG09589.1 	REAGCSMCLGMNPDILAPKERCASTSNRNFEGRQGALSRTHLMSPVMAAAAALVGKLADV
AN5886.1  	REAGCSMCLGMNPDILSPQERCASTSNRNFEGRQGAGGRTHLMSPAMAAAAAIVGKLADV
          	****************:*:***************** .*******.*****.:*******

MG01553.1 	RKLTDYKASPHIAAY---QKSTVTKPHVDERINQDAHEKDIIADIPEDNNGPHTNTSASV
NCU04385.1	RKLTDYKSSPHVEAAVIPETTSTAKAHTDERIEEDDVEKDKLADQPQDSS-PQVNTSVSK
FG09589.1 	RKLSHYTHQSAFKAR----ELPAEEPHVDERTKDDSEERELIGDQPQDSE-PHTNNLVSA
AN5886.1  	R--EHIAESPRLGKV-----QPKVDVKPEAEDVDTEEELDHILDQPADNE-PHTNTHTPA
          	*   .   .. .         .  . : : .  :   * : : * * *.. *:.*. .. 

MG01553.1 	G-TSAGLPKFTILKGIAAPLEKANVDTDAIIPKQFLKTIKRTGLGNALFYEMRFNEDGTE
NCU04385.1	--SSAGLPKFTNLKGIAAPMEKANIDTDAIIPKQFLKTIKRTGLGSALFYEMRYNPDGSE
FG09589.1 	AGSASGLPKFTIWKGTAAALDRSNVDTDAIIPKQFLKTIKRTGLGTALFYELRYKEDGSE
AN5886.1  	T-TSAGLPKFTTLKGIAAPMDRSNVDTDAIIPKQFLKTIKRTGLGSALFYELRYK-DGQE
          	  :::******  ** **.::::*:********************.*****:*:: ** *

MG01553.1 	KSDFVLNKEPYRKASILVCTGANFGCGSSREHAPWALNDFGIRSVIAPSFADIFFNNSFK
NCU04385.1	NPNFVLNREPYRSAKIIVCTGANFGCGSSREHAPWALNDFGVKSVIAPSFADIFFNNSFK
FG09589.1 	DPDFVLNQEPFRNTKTLVVTGPNFGCGSSREHAPWALLDFGIKCIIAPSFADIFFNNTFK
AN5886.1  	DPSFILNQGIYRNSKILVVTGPNFGCGSSREHAPWALLDFGIKCVIAPSFADIFFNNTFK
          	...*:**:  :*.:. :* **.*************** ***::.:************:**

MG01553.1 	NGMLPIPIKDQAQIEAIAAEARAGKEIEVDLPNQLIKNATGETICTFEVEEFRKHCLVNG
NCU04385.1	NGMLPIAIKDQAALERVHAEAAAGREIEIDLPNQLIKDADGNTLCEFEVEEFRKHCLVNG
FG09589.1 	NGMLPIRIDNKDDLDAIAAEARANRDIEVDLPSQLIKNAAGETICSFDVEEFRKHCLVNG
AN5886.1  	NGMLPVVIPDQAVLEKIADEARAGREVEVDLVNQEIKDEAGNKLASFDVDAFRKHCLING
          	*****: * ::  :: :  ** *.:::*:** .* **:  *:.:. *:*: ******:**

MG01553.1 	LDDIGLTMQMEDKIAEFEAKMTRETPWLDGTGYLKRKGQGGKLAAKAVPVPTTNRGEEKK
NCU04385.1	LDDIGLTMQMEDKIAEYEAKMSQQTPWLDGRAYLKRKGQGGKLVAKAVPVPKTNRGEEKK
FG09589.1 	LDDIGLTMQQEEKISEFERSMTKNTPWLDGSGYLKRPGQGGKLAAKAVPIPKTNRGEEKK
AN5886.1  	LDDIGLTLQMEDKIAKFEAKRTLDTPWLDGKAYLKRGRTGGSNMVKAAPVPKTNRGDVKG
          	*******:* *:**:::* . : :****** .****   **.  .**.*:*.****: * 

MG01553.1 	EPLEW
NCU04385.1	EPLEW
FG09589.1 	EPLEW
AN5886.1  	EPLEW
          	*****
```
